# Capnography for Assessing Nocturnal Hypoventilation and Predicting Compliance with Subsequent Noninvasive Ventilation in Patients with ALS

**DOI:** 10.1371/journal.pone.0017893

**Published:** 2011-03-30

**Authors:** Sung-Min Kim, Kyung Seok Park, Hyunwoo Nam, Suk-Won Ahn, Suhyun Kim, Jung-Joon Sung, Kwang-Woo Lee

**Affiliations:** 1 Department of Neurology, Seoul National University College of Medicine, Seoul, Korea; 2 Department of Neurology, Seoul National University, Bundang Hospital, Gyeonggi, Korea; 3 Department of Neurology, Boramae Hospital, Seoul, Korea; 4 Department of Neurology, Chung-Ang University Hospital, Seoul, Korea; 5 Department of Neurology, National Cancer Center, Gyeonggi, Korea; University of Giessen Lung Center, Germany

## Abstract

**Background:**

Patients with amyotrophic lateral sclerosis (ALS) suffer from hypoventilation, which can easily worsen during sleep. This study evaluated the efficacy of capnography monitoring in patients with ALS for assessing nocturnal hypoventilation and predicting good compliance with subsequent noninvasive ventilation (NIV) treatment.

**Methods:**

Nocturnal monitoring and brief wake screening by capnography/pulse oximetry, functional scores, and other respiratory signs were assessed in 26 patients with ALS. Twenty-one of these patients were treated with NIV and had their treatment compliance evaluated.

**Results:**

Nocturnal capnography values were reliable and strongly correlated with the patients' respiratory symptoms (*R* ^2^ = 0.211–0.305, *p* = 0.004–0.021). The duration of nocturnal hypercapnea obtained by capnography exhibited a significant predictive power for good compliance with subsequent NIV treatment, with an area-under-the-curve value of 0.846 (*p* = 0.018). In contrast, no significant predictive values for nocturnal pulse oximetry or functional scores for nocturnal hypoventilation were found. Brief waking supine capnography was also useful as a screening tool before routine nocturnal capnography monitoring.

**Conclusion:**

Capnography is an efficient tool for assessing nocturnal hypoventilation and predicting good compliance with subsequent NIV treatment of ALS patients, and may prove useful as an adjunctive tool for assessing the need for NIV treatment in these patients.

## Introduction

Amyotrophic lateral sclerosis (ALS) is a common neurodegenerative disease of adults with a prevalence of about 6 in 100,000. Most patients with ALS have progressive hypoventilation due to respiratory muscle weakness [1], which is associated with poor survival, cognitive impairment, and poor quality of life [2], [3], [4], [5]. Hypoventilation in these patients can easily worsen during sleep as a result of sleep-disordered breathing (apnea and hypopnea), hypotonia of the accessory respiratory muscles, weak diaphragm, supine position (orthopnea), and a possible dysfunction of the central drive for respiration [2], [5]. Nocturnal pulse oximetry monitoring, which is widely accepted to be an important screening tool for assessing the degree of nocturnal hypoventilation and the need for noninvasive ventilation (NIV) in patients with ALS [6], [7], [8], [9], assesses the oxygenation status rather than the ventilation status of patients. As such, it may not be sufficiently sensitive to detect nocturnal hypoventilation or to reveal the need for ventilatory support.

Capnography is a simple and noninvasive tool that enables the indirect but continuous monitoring of ventilatory status by measuring the end-tidal carbon dioxide (ETCO2) levels in the expired air [10]. It has been validated as a reliable screening method for sleep apnea syndromes [11] and an indicator of arterial partial pressure of carbon dioxide (*P*aCO2) in most spontaneously breathing adult patients [12], and it is strongly correlated with transcutaneous carbon dioxide (tcCO2) data in pediatric patients with sleep-disordered breathing [13].

The aim of this study was to determine the efficacy of capnography monitoring for assessing nocturnal hypoventilation and for predicting compliance with subsequent NIV treatment in ALS patients.

## Methods

### 1. Patients

This study was conducted at the Seoul National University ALS clinic between March 2006 and July 2009. Twenty-six patients (19 males) with either definite or probable ALS [14] who had subjective symptoms associated with hypoventilation (orthopnea, dyspnea on exertion, daytime drowsiness, morning headache, unrefreshing sleep, or sleep fragmentation due to shortness of breath) or decreased vital capacity (<80% of normal) were studied [15], [16]. Exclusion criteria were a pulmonary disease at inclusion, including pneumonia, history of NIV treatment or oxygen supplementation, or a tracheostomy at inclusion [12], [17], [18]. (Table 1). This study was approved by the institutional review board (IRB) of Seoul National University Hospital, and written informed consent was obtained from all participants.

**Table 1:** Baseline characteristics of the amyotrophic lateral sclerosis (ALS) patients.

Demographics	
*n*	26
Age, years	53.92±9.83 (38–74)
Males∶females	19∶7
ALSFRSr	32.00±8.85 (20–47)
Disease duration, months	24.51±8.10 (8.10–49.5)
VC, % predicted	57.19±27.71 (8–104)
Respiratory symptoms, *n* (%)	
Orthopnea	21 (80.77%)
Daytime drowsiness	21 (80.77%)
Dyspnea on exertion	18 (69.2%)
Subjective sleep fragmentation due to shortness of breath	3 (11.5%)
ABGA	
pH	7.41±0.04 (7.306–7.47)
PaCO_2_, mmHg	45.43±10.62 (36.6–83.8)
PaO_2_, mmHg	92.81±19.22 (61.2–130)
HCO_3_^–^, mmol/l	27.29±3.89 (24.0–41.0)
SaO_2_, %	96.41±1.73 (93.1–99.2)
Severity of nocturnal hypercapnea/hypoxia	
Percentage of total sleep time with ETCO_2_>47 mmHg	16.55±31.04 (0–93.98)
Maximal nocturnal ETCO_2_, mmHg	54.32±12.57 (38–84)
Average nocturnal ETCO_2_, mmHg	42.07±9.01 (23–63.91)
Percentage of total sleep time with *S*aO_2_<90%	8.98±21.08 (0–94.29)
Minimal nocturnal *S*aO_2_, %	80.20±19.32 (1–93)
Average nocturnal *S*aO_2_, %	94.49±3.03 (84.04–97)
Bulbar function	
Bulbar subscores in ALSFRSr	5.77±2.20 (0–8)
Patients with severe bulbar dysfunction, number (percent)	4, (15.4%)
Compliance with NIV, hours/day (applied in 21 patients)	
	4.74±2.93 (0–10)

Except where stated otherwise, data are expressed as mean±standard deviation (range) values. ALSFRSr, ALS Functional Rating Scale-Revised; ABGA, arterial blood gas analysis; ETCO_2_, end-tidal carbon dioxide; NIV, noninvasive ventilation; VC, vital capacity; *S*aO_2_, arterial oxygen saturation; *P*aO_2_, arterial partial pressure of oxygen; *P*aCO_2_, arterial partial pressure of carbon dioxide.

### 2. Parameters

Capnography (CO2SMO, Respironics Novametrix, Wallingford, CT, USA) with a nasal prong [11] was used to measure the duration of nocturnal hypercapnea during sleep (defined as the percentage of the total sleep time when ETCO2>47 mmHg) [19]. the average ETCO2 during sleep, and the maximal nocturnal ETCO2. Pulse oximetry (CO2SMO, Respironics Novametrix) was used to measure the duration of nocturnal hypoxia during sleep (assessed both as the percentage of the total sleep time when arterial oxygen saturation, *S*aO2, <90% and when *S*aO2<95%) [3], [7], the average pulse oximetry level, and the minimal nocturnal *S*aO2. Nocturnal vital signs, such as respiration rate and pulse rate, were also measured with capnography and pulse oximetry. Daytime arterial blood gas analysis while in the supine position, disease severity, and the degree of respiratory difficulty (ALS Functional Rating Scale–Revised, ALSFRSr) were also analyzed [20]. Analysis of the presence and severity of hypercapnea during sleep in patients with ALS was performed using NovaCARD software (Respironics Novametrix). Brief waking supine monitoring of capnography and oximetry were also performed while the patients were in a supine position for more than 10 min. Scores on a nocturnal-orthopnea questionnaire as part of the ALSFRSr were used as measures of symptoms of nocturnal hypoventilation, and sums of ALSFRSr scores for ‘speech’, ‘salivation’, and ‘swallowing’ as part of the ALSFRSr were used as measures of bulbar function [20].

### 3. NIV treatment

After obtaining informed their consent to participate, patients who had orthopnea [8] or who met the published criteria of Leigh *et al.* [16] were offered NIV in the hospital during admission, using a bilevel positive-pressure ventilator (BiPAP; Respironics, Vitalaire, Italy). Titration of NIV was achieved during admission as follows: initial low NIV pressure [inspiratory positive-airway pressure/expiratory positive-airway pressure (IPAP/EPAP) of 8/4 cmH2O] in the S/T mode was used to adjust the NIV, and then the NIV and IPAP/EPAP were titrated until respiratory symptoms (dyspnea or orthopnea) disappeared and/or a tidal volume of 10–15 ml/kg [21] was delivered to patients in a comfortable condition.

The degree of compliance with the NIV treatment was defined according to the mean NIV use per day and assessed in the outpatient clinic or at the patient's home at 1 month after treatment [8]. According to previous studies, good compliance with NIV treatment can be defined as NIV use for more than 4 hours per day [22], [23].

### 4. Statistical analysis

Linear regression analysis and nonparametric receiver operating characteristic (ROC) curve statistical analyses were performed with Predictive Analytics Software (version 18); a significance level of *p*<0.05 was used throughout.

## Results

### 1. Clinical and demographic characteristics

The age, respiratory symptoms and signs, disease severity, and compliance with subsequent NIV treatment of the included patients are given in Table 1.

### 2. Reliability of capnography and its correlation with nocturnal hypoventilation symptoms

**Figure 1:**
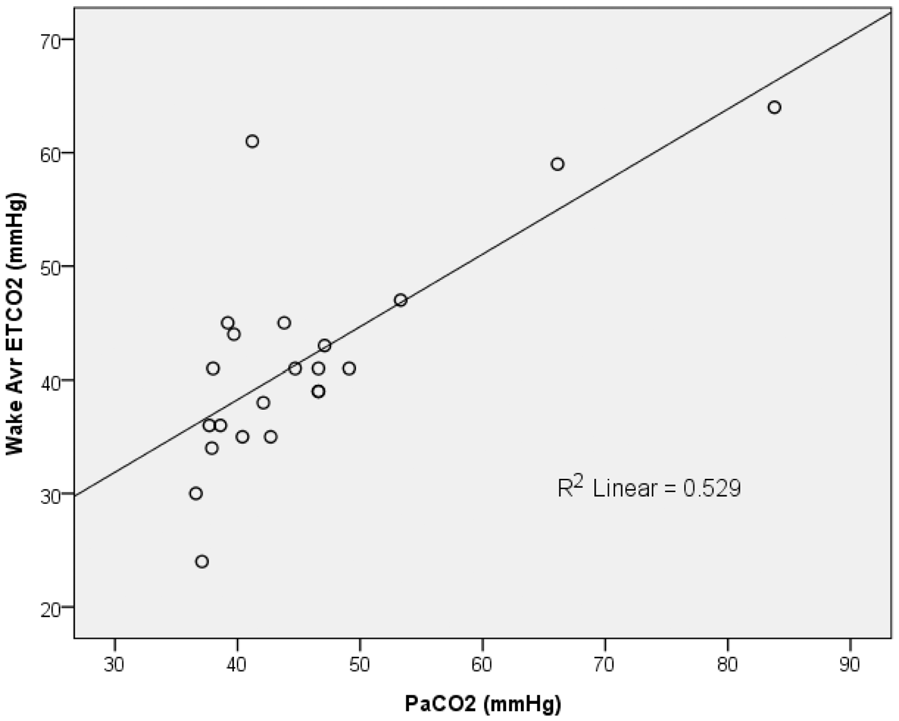
The average level of end-tidal carbon dioxide (ETCO_2_) measured by waking supine capnography correlated significantly with arterial partial pressure of carbon dioxide (*P*aCO_2_) level, as measured by arterial blood gas analysis (*p*<0.001).

**Figure 2:**
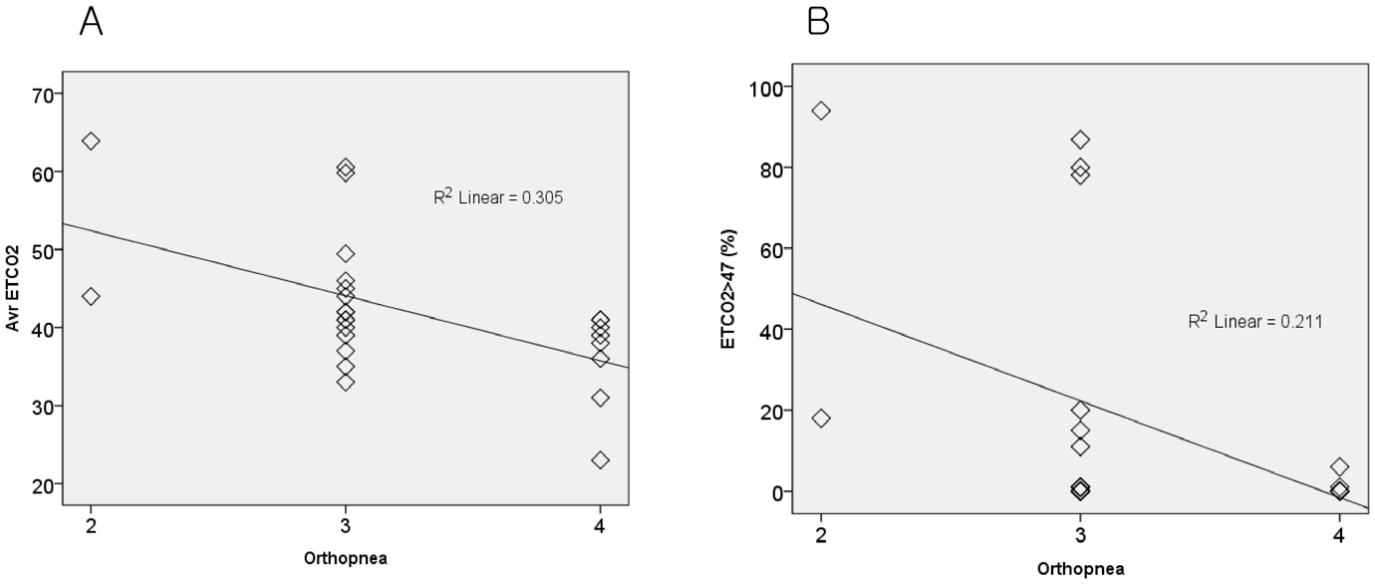
Correlations between nocturnal respiratory symptoms (orthopnea) with respiratory monitoring results. Scores from orthopnea questionnaire were strongly correlated with the average level of ETCO_2_ (*R* ^2^ = 0.305, *p* = 0.004) (A) and the duration of hypercapnea measured by nocturnal capnography (*R* ^2^ = 0.211, *p* = 0.021) (B).

Nocturnal and waking supine ETCO2 data were obtainable in 25 patients, with the remaining one patient continuing mouth breathing. Of those 25 patients, the waking supine ETCO2 was strongly correlated with their *P*aCO2, implying that capnography was a reliable screening tool for measuring *P*aCO2 in our patients (Figure 1). Both nocturnal average ETCO2 (Figure 2-A) and the duration of nocturnal hypercapnea (Figure 2-B) were strongly correlated with the severity of nocturnal respiratory symptoms (orthopnea-questionnaire scores from the ALSFRSr; Figure 2). However, the respiratory signs among patients with the same orthopnea-questionnaire scores varied markedly. For example, among patients with a score on the orthopnea questionnaire of 3, the average ETCO2 ranged from 33 to 61 mmHg (Figure 2-A) and the duration of nocturnal hypercapnea ranged from 0% to 87% of the total sleep time (Figure 2-B), which means that our assessment of subjective nocturnal respiratory symptoms may not have been sufficiently sensitive to reflect the actual degree of nocturnal hypoventilation or the need for ventilatory support in these patients (Figure 2).

### 3. Prediction of good compliance with subsequent NIV treatment with nocturnal respiratory signs, respiratory symptoms, and bulbar function

**Figure 3:**
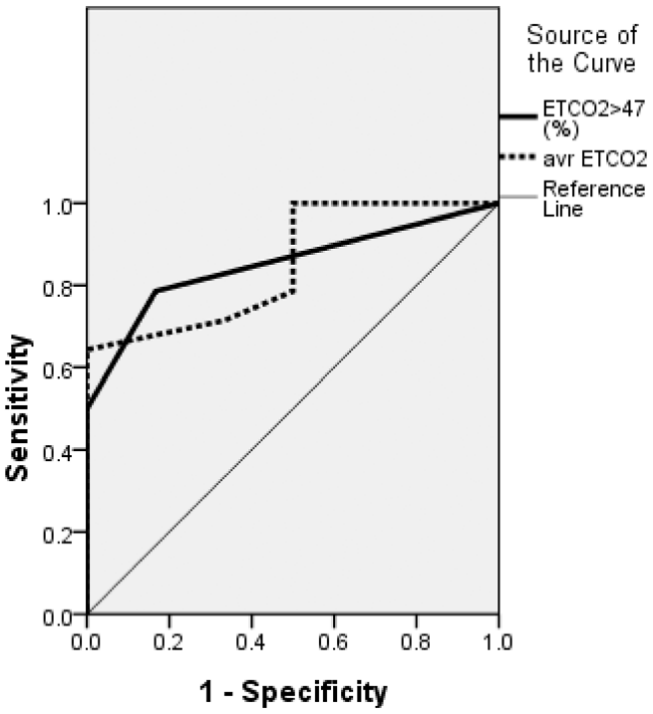
Receiver operating characteristic (ROC) curve for the duration of nocturnal hypercapnea relative to the total sleep time (ETCO_2_>47%) and the average nocturnal ETCO_2_ level (avr ETCO_2_) as predictors of good compliance with subsequent noninvasive ventilation (NIV) treatment. The areas under the curve (AUCs) were 0.846 (95% confidence interval, CI: 0.628–1.000) and 0.856 (95% CI: 0.00–1.000), respectively.

ROC curve analysis revealed good predictive values for compliance with subsequent NIV treatment for the duration of nocturnal hypercapnea (ETCO2>47 mmHg) and average nocturnal ETCO2, with area-under-the-curve values of 0.846 and 0.853, respectively. However, no significant findings were obtained in this regard for the duration of nocturnal hypoxia, average level of nocturnal *S*aO2, and ALSFRSr scores for orthopnea and bulbar function (Figure 3, Table 2, Figure S1). The duration of nocturnal hypercapnea during sleep had a sensitivity of 78.6% and a specificity of 83.3% for predicting good compliance with subsequent NIV treatment with a cut-off value of more than 1% of the total sleep time, and had a sensitivity of 50% and a specificity of 100% with a cut-off value of more than 5% of the total sleep time (Table S1).

**Table 2:** Receiver operating characteristic (ROC) curve analysis for parameters obtained by monitoring with nocturnal capnography and pulse oximetry, scores on an orthopnea questionnaire, and scores on a bulbar-function questionnaire as predictors of good compliance with subsequent NIV treatment.

Parameter	AUC	*p*	95% Confidence interval
**Duration for ETCO_2_>47 mmHg**	**0.846**	**0.018**	**0.628–1.000**
**Average ETCO_2_**	**0.853**	**0.016**	**0.000–1.000**
Duration for *S*aO_2_<95%	0.596	0.511	0.328–0.864
Average *S*aO_2_	0.417	0.569	0.147–0.687
Orthopnea questionnaire	0.218	0.054	0.000–0.466
Bulbar-function questionnaire	0.429	0.630	0.125–0.734

AUC: area under the curve.

### 4. Brief waking supine capnography and pulse oximetry monitoring as a screening tool for hypoventilation

**Figure 4:**
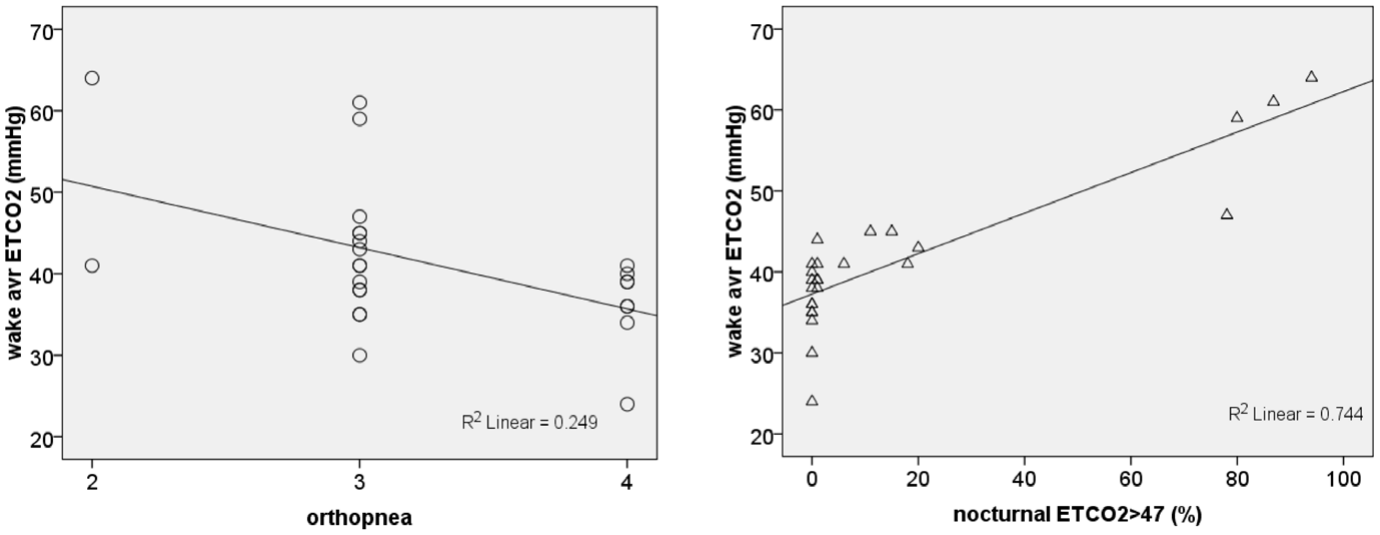
The average ETCO_2_ level obtained with brief wake capnography screening (wake avr ETCO_2_) correlated significantly with symptoms of patients for nocturnal hypoventilation (orthopnea; *p* = 0.021) (A) and duration of nocturnal hypercapnea relative to the total sleep time (nocturnal ETCO_2_>47%; *p*<0.001) (B).

**Figure 5:**
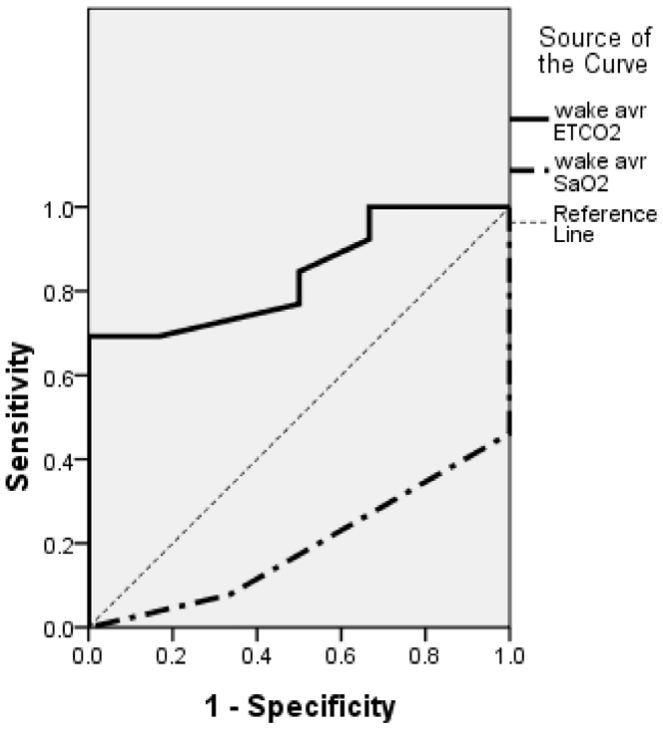
ROC curves for average ETCO_2_ measured by wake capnography screening and average *S*aO_2_ measured by wake pulse oximetry screening as predictors of good compliance with subsequent NIV treatment. The AUC values were 0.840 (95% CI: 0.628–1.000) and 0.192 (95% CI: 0.000–0.389), respectively.

While nocturnal capnography is a simple method of measuring nocturnal respiratory insufficiency, it requires continuous overnight monitoring. Thus, we also evaluated the efficacy of shorter-duration daytime waking supine capnography monitoring (mean duration, 20.58 min). The average level of waking supine ETCO2 was significantly correlated with the duration of nocturnal hypercapnea and nocturnal respiratory symptoms in our patients (Figure 4). ROC curve analysis revealed a high diagnostic power of average ETCO2 level measured by waking supine capnography screening as a predictor of good compliance with subsequent NIV treatment, but not of *S*aO2 measured by waking supine pulse oximetry screening (Figure 5). With a cut-off mean ETCO2 value of more than 39.50 mmHg, the brief waking capnography screening exhibited a sensitivity of 69.2% and a specificity of 83.3% for predicting good compliance with subsequent NIV treatment (Table S2).

## Discussion

We have shown herein that capnography can reflect *P*aCO2 level reliably, that it is strongly correlated with nocturnal respiratory symptoms and, moreover, that it is highly predictive of good compliance with subsequent NIV treatment in selected patients with ALS. In addition, brief waking supine capnography can be useful as a screening tool for nocturnal hypoventilation and compliance with subsequent NIV treatment.

Early detection and correction of hypoventilation is important in patients with ALS, because it can slow disease progression and improve cognitive function and quality of life [3], [4], [5], [8], [24]. However, because NIV treatment that is initiated too early can cause poor compliance due to discomfort in some patients [8], and since patients with ALS can benefit only from sufficient use of NIV (NIV compliance >4 hours/day) [23], correctly selecting patients who are really in need of NIV treatment is also important.

Several previous studies that investigated the efficacy of measuring ETCO2 as an indicator of *P*aCO2 found that it can only be accepted as an accurate predictor of *P*aCO2 when the following clinical characteristics apply [11], [12], [17], [18], [22]:

1. Intubated patients [17].
2. Spontaneously breathing (nonintubated) patients without significant dead space in the lung [18], [25], NIV use, or oxygen supplements [12].
3. ETCO2 recorded with a nasal prong and not with a nasobuccal mask, which might lower the diagnostic sensitivity in patients with severe respiratory symptoms [11].

By including only patients conforming with the above conditions we showed that ETCO2 monitoring can be useful in selected patients with ALS.

The presence of nocturnal respiratory or sleep-related symptoms can be a good indication for initiating NIV in patients with ALS [8]. However, as shown in the present study, measurement of ETCO2 by capnography monitoring is another good supportive indication for NIV, and has a higher predictive power for identifying patients who are likely to comply well with subsequent NIV treatment than either pulse oximetry monitoring or assessment of subjective symptoms with ALSFRSr (Table 2).

The orthopnea part of the ALSFRSr assesses symptoms for nocturnal respiratory difficulty and is graded as follows: 0, unable to sleep; 1, can only sleep sitting up; 2, needs extra pillows (more than two) in order to sleep; 3, some difficulty in sleeping at night due to shortness of breath, does not routinely use more than two pillows; and 4, no orthopnea during sleep [20]. However, ‘some difficulty in sleeping at night due to shortness of breath’ is too subjective and vague, so that patients with various degrees of respiratory signs can be included in this category, as was the case in our study (Figure 2). In addition, some patients with ALS prefer the decubitus position rather than elevating their head with extra pillows to compensate for their nocturnal hypoventilation [26], which implies that the number of pillows alone might not be a sensitive indicator for the degree of nocturnal hypoventilation.

While pulse oximetry has been widely used as a screening tool for nocturnal hypoventilation in ALS [6], [7], [8], [9] and can be useful for detecting patients who need a tracheostomy [7], its diagnostic power was insufficient for predicting compliance with subsequent NIV treatment in the present study. This might be because the main cause of nocturnal respiratory insufficiency in ALS is a ventilation impairment in which hypercapnea causes hypoxia, rather than an oxygenation impairment in which hypoxia precedes hypercapnea [10]. Therefore, measurement of their ventilatory status with capnography might be more effective in predicting NIV compliance than measurement of oxygenation status with pulse oximetry, as was shown in this study.

Brief waking supine capnography can address the effect of respiratory muscle weakness and orthopnea, which are supposed to play an important role in aggravating nocturnal hypoventilation [27]. This could explain its correlation with nocturnal respiratory symptoms and signs and its predictive value for good compliance with subsequent NIV treatment. In addition, it might also imply that these two factors can play a major role in determining the compliance with NIV treatment.

Compliance with NIV treatment in patients with ALS can be influenced by various factors, such as signs or symptoms of nocturnal hypoventilation, and the presence of bulbar symptoms [3], [8]. We assessed both the need for NIV (orthopnea-questionnaire scores, nocturnal hypercapnea, and nocturnal hypoxia) and treatment obstacles (ALSFRSr scores for speech, salivation, and swallowing). However, only the severity of hypercapnea as measured by capnography had a good predictive value for subsequent NIV compliance (Table 2). Our results suggest that the need for NIV in patients with ALS is more of an influence than an obstacle for compliance with subsequent NIV treatment.

The mean compliance to subsequent NIV in our study is relatively low (mean: 4.74 hours/day, ranged 0–10). This seems largely because patients who complain subjective orthopnea/dyspnea but who didn't have objective signs of nocturnal hypoventilation were also included. The significantly longer duration of nocturnal hypercapnea in good compliance group (n = 14), than that in poor compliance group (n = 6) (28.27±37.85% vs 0.17±0.41% respectively, p = 0.016) in this study also support our hypothesis for this phenomenon.

Our study was subject to a few limitations. First, our patient population was small. Second, we chose a BiPAP ventilator as a standard NIV treatment. This particular instrument was the only small portable NIV aid available in our clinic at the beginning of this study. Whether such instruments are adequate for patients with neuromuscular disease remains a matter of debate due to their variable ability to maintain lung compliance and provocation of coughing [28]. However, a recent randomized controlled study found that pressure and volume ventilators are equivalent in terms of their effects on physiology, function, and quality of life [29]. Third, the majority of patients enrolled in this study had relatively well preserved pulmonary function and were in their early stage of disease.(mean vital capacity was 57.19% of normal and mean ALSFRSr was 32.0) Our result may not be extrapolated to patients with ALS in more advanced stage of disease. Finally, we used ETCO2 to indirectly monitor nocturnal *P*aCO2, which has some inherent limited clinical indications (as described above) and prevented us from monitoring one of our patients with severe bulbar dysfunction and subsequent mouth breathing. tcCO2 may have been applicable to more patients.

In conclusion, we have shown that in selected patients with ALS, both nocturnal and brief waking supine capnography can play important roles in assessing nocturnal hypoventilation and can have a higher diagnostic value in predicting good compliers to subsequent NIV treatment than conventional pulse oximetry monitoring or assessment of subjective symptoms. Capnography can thus be useful as an adjunctive tool for assessing the need for NIV in patients with ALS.

## Supporting Information

Figure S1 **Receiver operating characteristic (ROC) curve for duration of hypoxia (arterial oxygen saturation,** ***S*** **aO_2_<95%), average nocturnal** ***S*** **aO_2_ level (avr** ***S*** **aO_2_), bulbar function (bulbar scores), and symptoms for nocturnal hypoventilation (orthopnea) as predictors of good compliance with subsequent noninvasive ventilation treatment.**
(TIF)

Table S1 **Sensitivities and specificities of nocturnal capnography, pulse oximetry, and scores from the orthopnea and bulbar function questionnaire in Amyotrophic Lateral Sclerosis Functional Rating Scale – Revised (ALSFRSr) for predicting good compliance with subsequent noninvasive ventilation (NIV) treatment are listed according to their cut-off values.** ETCO_2_, end-tidal carbon dioxide; avr, average; *S*aO_2_, arterial oxygen saturation.
(DOC)

Table S2 **Sensitivities and specificities of waking capnography and pulse oximetry for predicting good compliance with following NIV treatment are listed according to their cut-off values.**
(DOC)
