# Supplementary figures and images for: Capnography for Assessing Nocturnal Hypoventilation and Predicting Compliance with Subsequent Noninvasive Ventilation in Patients with ALS
Source: PLoS One. 2011 Mar 30;6(3):e17893. doi: 10.1371/journal.pone.0017893 (PMC3068132; doi:10.1371/journal.pone.0017893)

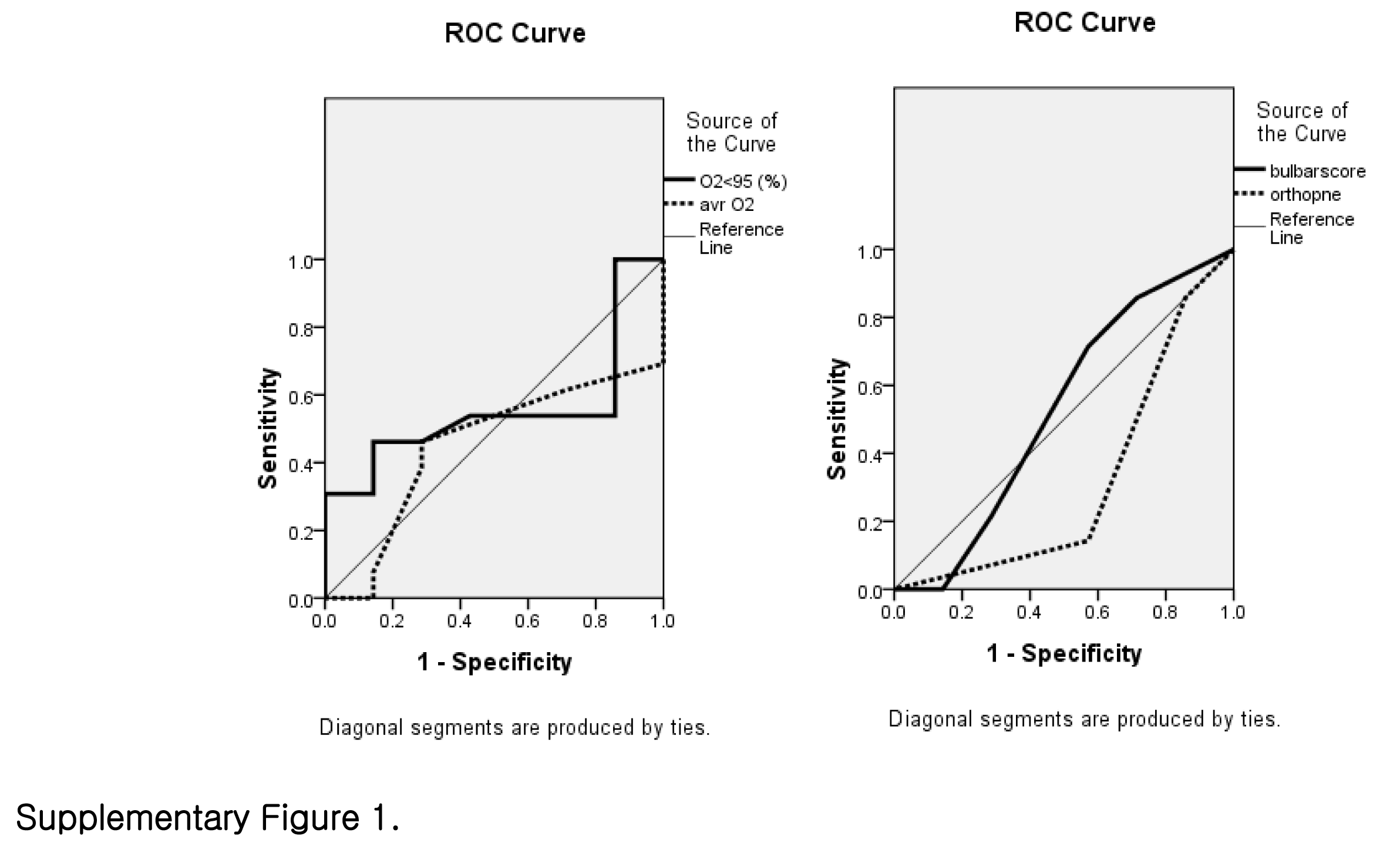

Supplement: Figure S1 — Receiver operating characteristic (ROC) curve for duration of hypoxia (arterial oxygen saturation, S aO2<95%), average nocturnal S aO2 level (avr S aO2), bulbar function (bulbar scores), and symptoms for nocturnal hypoventilation (orthopnea) as predictors of good compliance with subsequent noninvasive ventilation treatment. (TIF) [file pone.0017893.s001.tif]
