# Supplementary material for: Capnography for Assessing Nocturnal Hypoventilation and Predicting Compliance with Subsequent Noninvasive Ventilation in Patients with ALS
Source: PLoS One. 2011 Mar 30;6(3):e17893. doi: 10.1371/journal.pone.0017893 (PMC3068132; doi:10.1371/journal.pone.0017893)
Supplement: Table S1 — Sensitivities and specificities of nocturnal capnography, pulse oximetry, and scores from the orthopnea and bulbar function questionnaire in Amyotrophic Lateral Sclerosis Functional Rating Scale – Revised (ALSFRSr) for predicting good compliance with subsequent noninvasive ventilation (NIV) treatment are listed according to their cut-off values. ETCO2, end-tidal carbon dioxide; avr, average; SaO2, arterial oxygen saturation. (DOC) [file pone.0017893.s002.doc]

| Test result variable | Positive if greater than or equal to | Sensitivity | 1 – Specificity |
| --- | --- | --- | --- |
| ETCO_2_ >47 mmHg (%) | –1.00 | 1.000 | 1.000 |
|  | ***1.00*** | ***0.786*** | ***0.167*** |
|  | ***5.00*** | ***0.500*** | ***0.000*** |
|  | 16.50 | 0.462 | 0.000 |
|  | 19.00 | 0.385 | 0.000 |
|  | 49.02 | 0.308 | 0.000 |
|  | 79.01 | 0.231 | 0.000 |
|  | 83.40 | 0.154 | 0.000 |
|  | 90.41 | 0.077 | 0.000 |
|  | 94.98 | 0.000 | 0.000 |
| avr ETCO_2_ (mmHg) | 22.00 | 1.000 | 1.000 |
|  | 29.00 | 1.000 | 0.833 |
|  | 35.50 | 1.000 | 0.667 |
|  | 36.50 | 1.000 | 0.500 |
|  | 37.50 | 0.923 | 0.500 |
|  | 38.50 | 0.846 | 0.500 |
|  | 39.50 | 0.769 | 0.500 |
|  | 40.50 | 0.692 | 0.333 |
|  | 41.50 | 0.692 | 0.000 |
|  | 43.00 | 0.538 | 0.000 |
|  | 45.00 | 0.385 | 0.000 |
|  | 47.72 | 0.308 | 0.000 |
|  | 54.63 | 0.231 | 0.000 |
|  | 60.18 | 0.154 | 0.000 |
|  | 62.23 | 0.077 | 0.000 |
|  | 64.91 | 0.000 | 0.000 |
| *S*aO_2_ <95% (%) | 0.00 | 1.000 | 1.000 |
|  | 1.50 | 1.000 | 0.833 |
|  | 2.50 | 0.923 | 0.833 |
|  | 4.00 | 0.846 | 0.833 |
|  | 5.29 | 0.692 | 0.833 |
|  | 7.29 | 0.615 | 0.833 |
|  | 9.50 | 0.538 | 0.833 |
|  | 11.00 | 0.538 | 0.667 |
|  | 13.00 | 0.538 | 0.500 |
|  | 19.50 | 0.538 | 0.333 |
|  | 30.40 | 0.462 | 0.167 |
|  | 36.07 | 0.462 | 0.000 |
|  | 38.17 | 0.385 | 0.000 |
|  | 50.00 | 0.308 | 0.000 |
|  | 74.98 | 0.231 | 0.000 |
|  | 91.29 | 0.154 | 0.000 |
|  | 96.31 | 0.077 | 0.000 |
|  | 101.00 | 0.000 | 0.000 |
| avr *S*aO_2_ (%) | 83.04 | 1.000 | 1.000 |
|  | 86.02 | 0.923 | 1.000 |
|  | 88.62 | 0.846 | 1.000 |
|  | 91.39 | 0.769 | 1.000 |
|  | 93.78 | 0.692 | 1.000 |
|  | 94.50 | 0.615 | 0.833 |
|  | 95.34 | 0.462 | 0.333 |
|  | 95.84 | 0.385 | 0.333 |
|  | 96.31 | 0.077 | 0.167 |
|  | 96.81 | 0.000 | 0.167 |
|  | 98.00 | 0.000 | 0.000 |
| Orthopnea | 1.00 | 1.000 | 1.000 |
|  | 2.50 | 0.846 | 1.000 |
|  | 3.50 | 0.154 | 0.667 |
|  | 5.00 | 0.000 | 0.000 |
| Bulbar score | 2.0000 | 1.000 | 1.000 |
|  | 5.5000 | 0.923 | 1.000 |
|  | 8.5000 | 0.846 | 0.833 |
|  | 9.5000 | 0.692 | 0.667 |
|  | 10.5000 | 0.154 | 0.333 |
|  | 11.5000 | 0.000 | 0.167 |
|  | 13.0000 | 0.000 | 0.000 |
| **Supplementary Table S1.** | | | |
|  | | | |
