# Supplementary material for: Capnography for Assessing Nocturnal Hypoventilation and Predicting Compliance with Subsequent Noninvasive Ventilation in Patients with ALS
Source: PLoS One. 2011 Mar 30;6(3):e17893. doi: 10.1371/journal.pone.0017893 (PMC3068132; doi:10.1371/journal.pone.0017893)
Supplement: Table S2 — Sensitivities and specificities of waking capnography and pulse oximetry for predicting good compliance with following NIV treatment are listed according to their cut-off values. (DOC) [file pone.0017893.s003.doc]

| **Coordinates of the curve** | | | |
| --- | --- | --- | --- |
| Test result variable | Positive if greater than or equal to | Sensitivity | 1 – Specificity |
| Wake avr ETCO_2_(mmHg) | 23.00 | 1.000 | 1.000 |
|  | 27.00 | 1.000 | 0.833 |
|  | 32.50 | 1.000 | 0.667 |
|  | 35.50 | 0.923 | 0.667 |
|  | 37.00 | 0.846 | 0.500 |
|  | 38.50 | 0.769 | 0.500 |
|  | ***39.50*** | ***0.692*** | ***0.167*** |
|  | 40.50 | 0.692 | 0.000 |
|  | 42.00 | 0.462 | 0.000 |
|  | 44.00 | 0.385 | 0.000 |
|  | 46.00 | 0.308 | 0.000 |
|  | 53.00 | 0.231 | 0.000 |
|  | 60.00 | 0.154 | 0.000 |
|  | 62.50 | 0.077 | 0.000 |
|  | 65.00 | 0.000 | 0.000 |
| Wake avr *S*aO_2_(%) | 46.00 | 1.000 | 1.000 |
|  | 69.00 | 0.923 | 1.000 |
|  | 93.00 | 0.769 | 1.000 |
|  | 95.50 | 0.462 | 1.000 |
|  | 96.50 | 0.077 | 0.333 |
|  | 98.00 | 0.000 | 0.000 |

**Supplementary Table S2.**
